# No difference of survival between cruciate retaining and substitution designs in high flexion total knee arthroplasty

**DOI:** 10.1038/s41598-021-85892-1

**Published:** 2021-03-22

**Authors:** Gun-Woo Kim, Quan He Jin, Jun-Hyuk Lim, Eun-Kyoo Song, Jong-Keun Seon

**Affiliations:** Department of Orthopaedic, Center for Joint Disease, Chonnam National University Hwasun Hospital and Medical School, Jeonnam, Republic of Korea

**Keywords:** Anatomy, Diseases, Health care, Medical research

## Abstract

The aim of this study was to compare the long-term implant survival and outcomes in patients with high-flexion cruciate-retaining (CR) or high-flexion posterior cruciate-substituting (PS) knee implants. A total of 253 knees (CR group: 159 vs. PS group: 94) were available for examination over a mean follow-up of 10 years. Clinical outcomes were assessed including the Hospital for Special Surgery score, Knee Society score and Western Ontario and McMaster Universities Osteoarthritis Index score at the final follow-up. Radiologic measurements were also assessed including the hip-knee-ankle angle and radiolucent lines according to the KSS system at the final follow-up. The survival rate was analyzed using the Kaplan–Meier method. At the final follow-up, the mean total HSS scores were similar between the two groups (*p* = 0.970). The mean hip-knee-ankle angle at the final follow-up was similar between groups (*p* = 0.601). The 10- and 15-year survival rates were 95.4% and 93.3% in the CR group and 92.7% and 90.9% in the PS group, respectively, with no significant difference. Similar clinical and radiographic outcomes could be achieved with both the high-flexion CR and high-flexion PS total knee designs without a difference in survival rate after a 10-year follow-up.

## Introduction

The main goals of total knee arthroplasty (TKA) are to achieve pain-free knees and good implant survival after the operation. More recently, increasing patient demands and expectations have led to the requirement for achieving higher flexions (approximately > 120°) after TKA^1^. To meet the patients’ expectation for a high range of motion (ROM), the high-flexion prosthesis design (NexGen LPS-Flex; Zimmer Inc., Warsaw, IN, USA) was introduced in TKA. These high-flexion designs are not radically different from their conventional design counterparts. Rather, they incorporate subtle changes in the geometry of components to allow improved contact area at high-flexion angles and thereby increase the posterior femoral translation in the high-flexion ranges compared with the conventional designs^2,3^.

Although there have been claims that the design features of high-flexion prostheses improve knee flexion, this has not been documented clinically, to the best of our knowledge. In addition, with the design of these new high-flexion TKA prostheses, new questions arise about the patterns of motion, forces, and stresses experienced by patients receiving these designs. Recently, several studies reported an occurrence of early radiolucent line around the femoral component related to repetitive deep flexion provided by high-flexion posterior-stabilized TKA in short term follow up^4,5^. However, only a few studies have compared outcomes between the high-flexion PS and high-flexion CR designs^6^. Furthermore, in the previous studies, evaluations were performed only in a small number of selected patients and short term follow up.

The aim of this study was to compare high-flexion CR and high-flexion PS prostheses in terms of complications including aseptic loosening and radiolucent lines, clinical and radiologic outcomes, and long-term survival rate after a mean follow-up of 10 years. The hypothesis was that there are no significant differences in outcomes between high-flexion PS knee implant and the high-flexion CR knee implant after a long-term follow-up.

## Materials and methods

We conducted a retrospective analysis of prospectively collected data on primary knee arthroplasties (TKAs) using high-flexion CR (NexGen CR-Flex; Zimmer Inc., Warsaw, IN, USA) or high-flexion PS prosthesis (NexGen LPS-Flex, Zimmer Inc.) performed at Chonnam National University Hwasun Hospital. Since the data were obtained from the retrospective study design, an informed consent was not needed according to the ethics committee of Chonnam National University Hwasun Hospital institutional review board. All methods were performed based on the relevant guidelines and regulations, and all experimental protocols that we used were approved by Chonnam National University Hwasun Hospital institutional review board.

**Figure 1:**
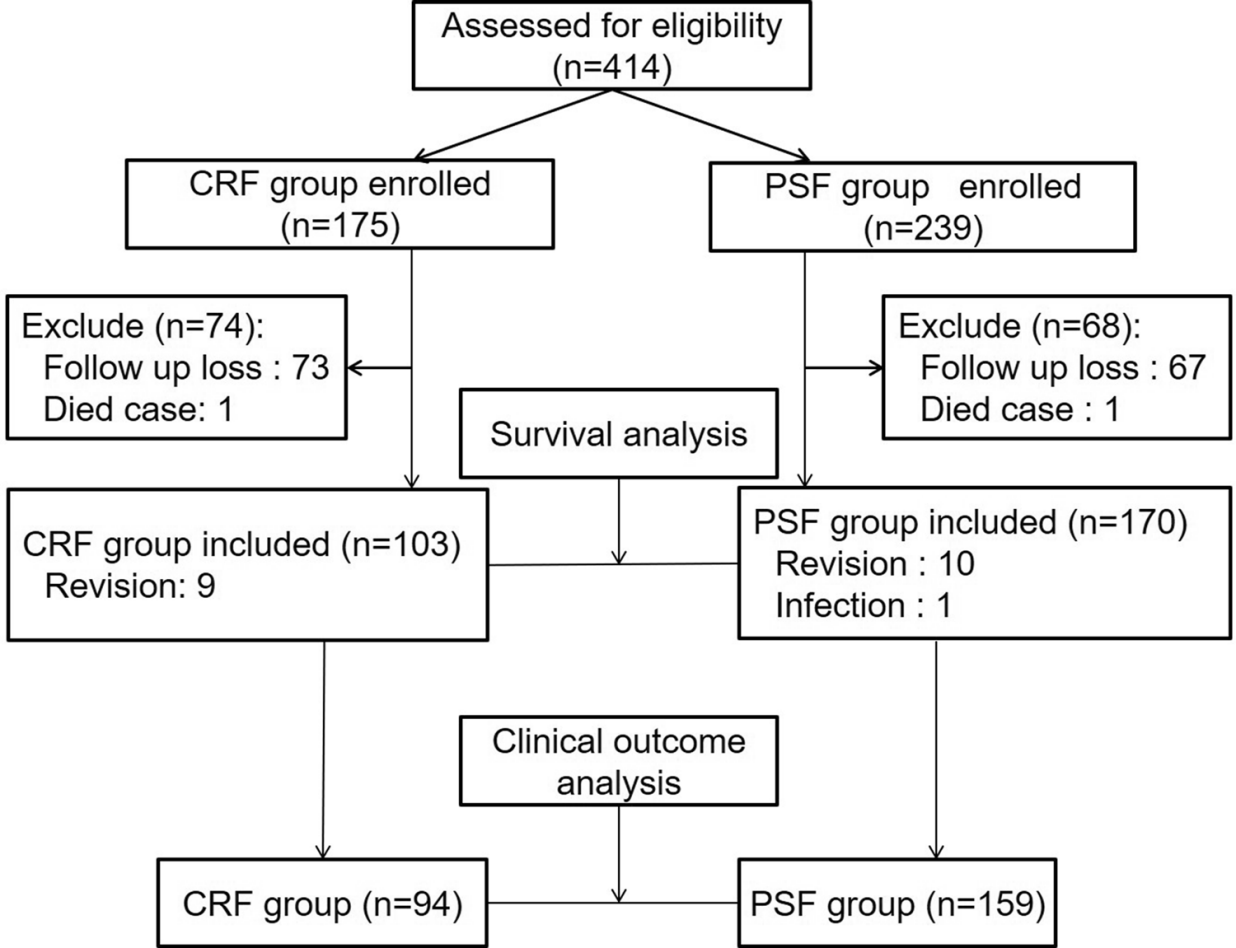
Flow diagram of the patient enrollment procedure, prepared according to CONSORT guidelines.

All patients who underwent TKA at our institution were enrolled in a prospective data registry. In our institution, all patients are treated in accordance with a highly standardized regimen, and clinical data are collected prospectively. From January 2000 to December 2010, a total of 414 consecutive primary TKAs among 367 patients were identified for the study. Two senior surgeons performed all the TKA procedures using the same technique. Patients with a history of open knee surgery, those with a severe deformity of the knee joint (varus alignment > 20°, flexion contracture > 30°, and valgus alignment > 10°), those with a knee joint disease other than primary osteoarthritis, and those undergoing revision TKA for diseases other than primary osteoarthritis were excluded. During the follow-up, 120 patients (140 knees) were unable to be contacted, and 2 patients died. Furthermore, patients who underwent revision or experienced other complications severely affecting the clinical outcomes were also excluded. Finally, the cohort included 143 patients (159 knees) in the CR group and 87 patients (94 knees) in the PS group for the analysis of long-term results (Fig. 1). The minimum follow-up duration was 8 years in this study (mean, 11.1 years; range, 8–17 years).

The CR group consisted of 10 men and 133 women with a mean age of 69.6 ± 7.1 years (range, 53–91 years) at the time of surgery. The PS group consisted of 5 men and 82 women with a mean age of 68.3 ± 7.2 years (range, 52–87 years) at the time of surgery. No significant differences were found between the two groups in terms of age (*p* = 0.206) and sex (*p* = 0.711) at the time of surgery. The mean preoperative non-weight-bearing ROM, Hospital for Special Surgery (HSS) score, Knee Society score (KSS, pain and function subscales), and Western Ontario and McMaster Universities Osteoarthritis Index (WOMAC) score were similar between the two groups (Table 1).

**Table 1:** Comparison of preoperative demographic data and clinical outcomes of the 230 patients (253 knees).

Parameters	CRF (N = 143)	PSF (N = 87)	*p*-value
Mean age (yr)	69.6 ± 7.1	68.3 ± 7.2	0.206*
Sex (M/F)	10 / 133	5 / 82	0.711†
Mean follow-up duration (y)	10.7 ± 1.6	11.6 ± 2.6	0.125‡
Range of Motion (deg)	123.9 ± 16.0	122.8 ± 15.6	0.544‡
HSS score	58.4 ± 13.8	59.2 ± 11.9	0.970*
KSS pain score	21.7 ± 9.6	20.4 ± 9.5	0.332*
KSS function score	49.7 ± 16.6	47.9 ± 14.9	0.446*
WOMAC score	66.0 ± 16.3	67.3 ± 19.2	0.619*

*Independent t-test.

^†^Chi-square test.

^‡^Mann–Whitney Test.

We used the same surgical procedure in both groups, except for retaining the posterior cruciate ligament (PCL) in the CR group and sacrificing the PCL in the PS group. All TKAs were performed with a longitudinal midline skin incision, followed by a medial parapatellar arthrotomy after patellar eversion. Then, the incision was extended into the quadriceps tendon by approximately 3–4 cm. In both groups, the gap balance technique was used for bone cutting and selecting the prosthesis size. To restore the knee to its premorbid state, care was taken to ensure that the thickness of the bone and cartilage removed was equal to the thickness of the prosthesis in all TKAs. Tibial cuts were performed using an extramedullary guide, with a posterior slope of 7° in the CR group and 3° in the PS group. The intramedullary rod was set at 4–6° valgus cut for femoral alignment in all TKAs. We resected the distal femoral condyles first, followed by the posterior femoral condyles, to ensure that the thickness of the bone and cartilage removed was equal to the thickness of the femoral component to be inserted. We removed osteophytes by rongeur and performed patellar denervation using an electrocautery device, but neither of groups resurfaced the patella. All prostheses were fixed with tobramycin-laden cement (Simplex P; Stryker, Mahwah, NJ, USA).

Similar postoperative pain control and rehabilitation treatment were provided in both groups. In both groups, passive motion was not performed immediately after surgery, and rehabilitation protocols, including full-weight bearing, were followed on the first postoperative day.

In all patients, physical examination and knee scoring were performed before surgery, at 6 months and 1 year after surgery, and annually thereafter. At the final follow-up, all the clinical results, including ROM, KSS, and WOMAC score, were compared. All the data were collected and evaluated by a surgical assistant from the surgical team to control bias. Supine anteroposterior and lateral radiographs and standing anteroposterior scanograms were obtained preoperatively and at each follow-up. An independent observer who was blind to the clinical results evaluated the radiographic outcomes, such as hip-knee-ankle (HKA) angle and radiolucent lines. The position of radiolucent lines was identified according to the recommendation of the KSS system^7^. The intraclass correlation coefficient was used to assess the intra-observer reliability for the evaluation of the HKA angle and incidence of radiolucent line, with twice assessment differs by two weeks from each other (ranges between 0.81 and 0.87).

### Statistical analysis

A statistical power analysis was considered based on the sample size calculation performed in the previous study^8^. Detecting a difference of 7° (standard deviation ± 11.2°) in flexion between CR group and PS group, 41 patients per group provided the power of 0.8 and the confidence level of 0.05. This study included 143 patients in the CR group and 87 patients in the PS group, which was enough for the requirement.

Statistical analyses were performed using SPSS for Windows (release 11.0; SPSS Inc., Chicago, IL, USA). Differences in parametric data, such as functional outcomes, between the two groups were analyzed using the independent t-test. Non-parametric data, such as ROM and follow-up duration, were compared with the Mann–Whitney U-test. The chi-square test was used to evaluate the overall non-numeric data in the two groups. The cumulative survival rate at the 10- and 15-year follow-up evaluations was compared between the two groups using the log-rank test. Differences of *p* < 0.05 were considered statistically significant.

## Results

The average preoperative ROM in the CR and PS groups was 123.9 ± 16.0° and 122.8 ± 15.6°, respectively (*p* = 0.544) (Table 1). At the final follow-up, the average ROM was 131.1 ± 17.4° and 129.5 ± 14.8°, respectively (*p* = 0.223). The preoperative clinical scores showed no significant differences between the two groups (Table 1). The clinical results at the final follow-up had no statistical significant between the CR and PS groups regard of HSS score (*p* = 0.970), KSS pain score (*p* = 0.565), KSS function score (*p* = 0.125), and WOMAC score (*p* = 0.338). (Table 2).

**Table 2:** Clinical outcomes at the final follow-up.

Parameters	CRF (94 knees)	PSF (159 knees)	*p*-value
Range of motion (deg)	131.1 ± 17.4	129.5 ± 14.8	0.223‡
HSS score	89.7 ± 13.1	89.7 ± 9.8	0.970*
KSS pain score	45.7 ± 9.4	45.1 ± 8.9	0.565*
KSS function score	88.0 ± 17.5	84.5 ± 17.2	0.125*
WOMAC score	10.7 ± 12.3	12.3 ± 11.8	0.338*

*Independent t-test.

^‡^Mann–Whitney Test.

According to the radiologic results, the HKA angle was similar between the CR and PS groups preoperatively and at the final follow-up (*p* = 0.312 and *p* = 0.601, respectively) (Table 3). In the coronal and sagittal radiographs, radiolucent lines were identified according to the zones reported by Ewald^7^. A radiolucent line was found in the tibial compartment in 10 (6.3%) knees and in the femoral compartment in 11 (6.9%) knees in the CR group, and in the tibial compartment in 6 (6.4%) knees and in the femoral compartment in 10 (11.5%) knees in the PS group, with no significant difference between the groups (*p* = 0.538). In the CR group, the radiolucent line in the tibial compartment was in coronal zone 1 in nine cases and in sagittal zone 1 in one case. In the PS group, the radiolucent line in the tibial compartment was in coronal zone 1 in four cases and in sagittal zone 1 in two cases. No difference was found in the tibial-side radiolucent line between the CR and PS groups (*p* = 0.978). For the femoral compartment, six knees showed a radiolucent line in femoral zone 1 and five knees in femoral zone 4 in the CR group, while eight knees showed a radiolucent line in femoral zone 1, one knee in zone 2, and four knees in zone 4 in the CR group. No difference was observed in the femoral-side radiolucent line between the two groups (*p* = 0.257). (Table 3) No evidence of dislocation of bearing or massive osteolysis (lesions > 1 cm) was found. However, one case of aseptic loosening (progressive lucency of > 2 mm) was detected in the CR group at postoperative 12 years.

**Table 3:** Radiological outcomes (mean, range) in both groups of knee.

Parameters	CRF (94 knees)	PSF (159 knees)	*p*-value
**Hip-Knee-Ankle angle**			
Pre-operative (varus)	9.4 ± 4.7	10.2 ± 6.4	0.312*
Last follow-up (varus)	2.0 ± 2.8	2.2 ± 4.5	0.601*
Radiolucent line (overall, number, %)			0.538†
Tibial side	10 (6.3%)	6 (6.4%)	0.978†
**Coronal aspect**			
Zone 1	9	4	
Zone 2			
Zone 3			
Zone 4			
Zone 5			
**Sagittal aspect**			
Zone 1	1	2	
Zone 2			
Zone 3			
Femoral side	11 (6.9%)	10 (11.5%)	0.257†
**Sagittal aspect**			
Zone 1	6	8	
Zone 2		1	
Zone 3			
Zone 4	5	4	
Zone 5			

Negative values indicate valgus alignment.

*Independent t-test.

^†^Chi-square test.

**Figure 2:**
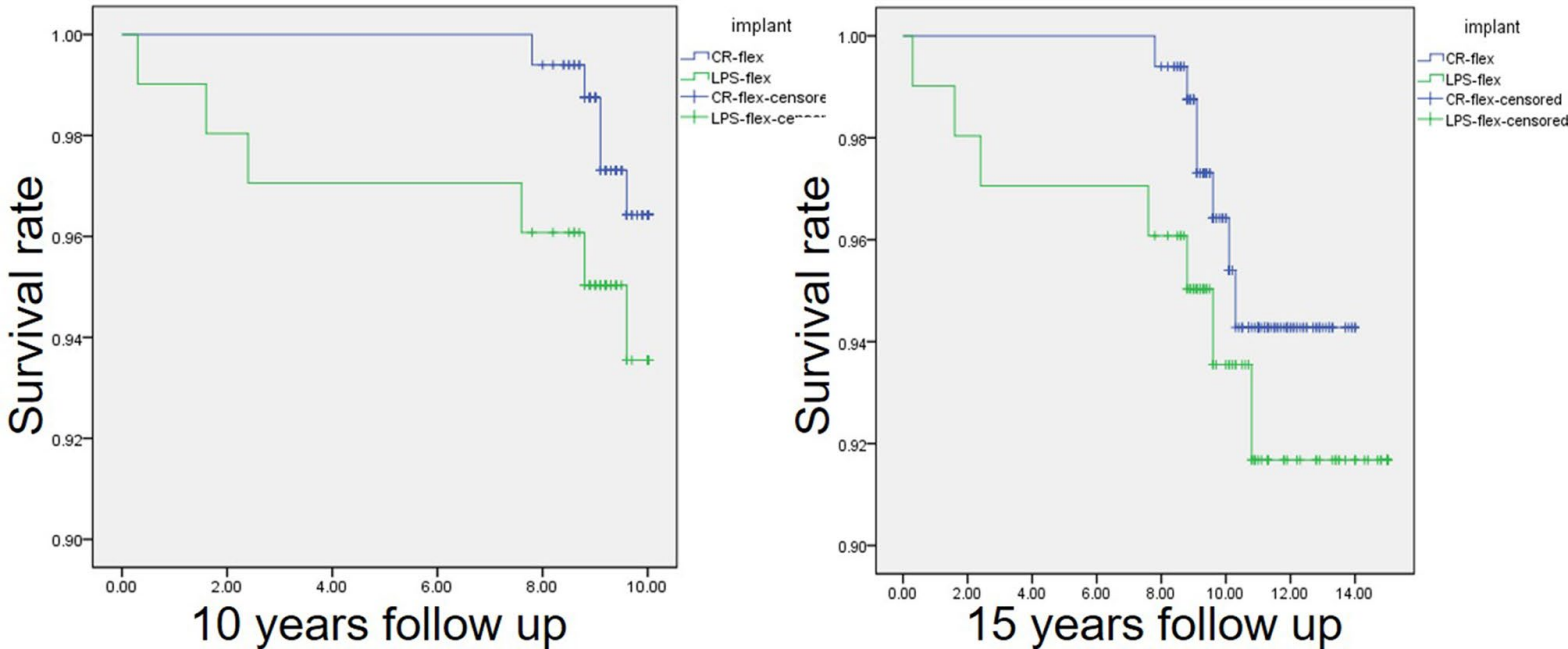
Survival rate of 10 years and 15 years follow up using Kaplan–Meier analysis.

**Figure 3:**
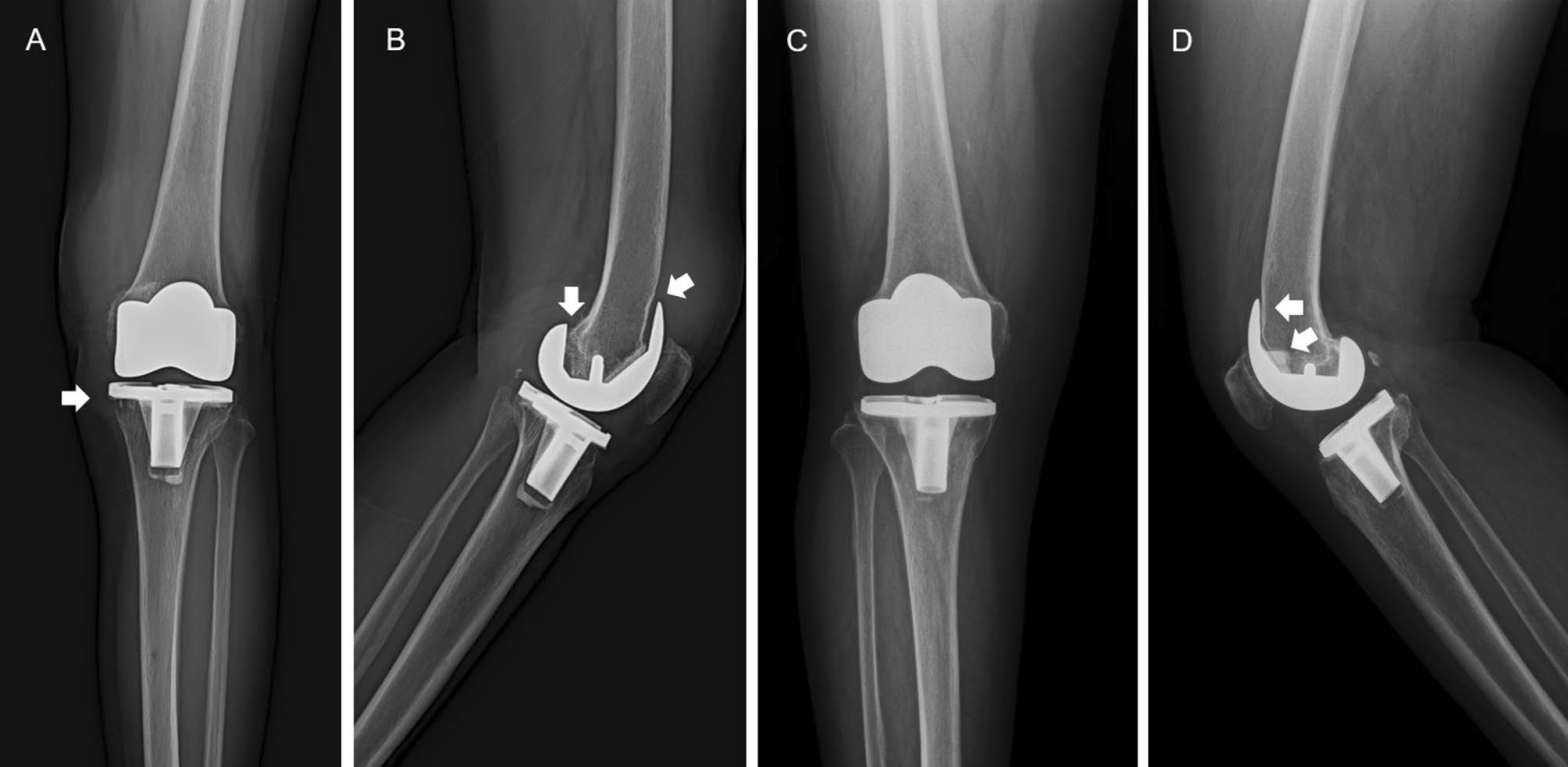
(**A**,**B**) Radiographs of Lt knee of a 70-year-old woman with taken 10 years after surgery revealing the NexGen high-flexion CR bearing prosthesis-fixed. (**A**) Anteroposterior radiograph. Radiolucent line is demonstrated zone 1 around tibial component according to the KSS system^7^. (**B**) Lateral radiograph. Radiolucent line is demonstrated zone 1, 4 around femoral component according to the KSS system. (**C**,**D**) Radiographs of Rt knee of a 72-year-old man with taken 10 years after surgery revealing the NexGen high-flexion LPS fixed-bearing prosthesis. (**A**) Anteroposterior radiograph. No radiolucent line is demonstrated around tibial component. (**B**) Lateral radiograph. Radiolucent line is demonstrated zone 1, 2 around femoral component according to the KSS system.

In the CR group, the estimated survival rate according to the Kaplan–Meier survival analysis was 96.4% (95% confidence interval: 96.1–96.6%) at 10 years, with an overall revision rate of 3.0% (5 of 166 knees), and 94.3% (95% confidence interval: 94.0–94.6%) at 15 years, with an overall revision rate of 4.2% (7 of 166 knees). In the PS group, the estimated survival rate was 93.5% (95% confidence interval: 93.0–94.0%) at 10 years, with an overall revision rate of 5.9% (6 of 102 knees) and 91.7% (95% confidence interval: 91.1–92.3%) at 15 years, with an overall revision rate of 4.2% (7 of 102 knees). In the Kaplan–Meier survival analysis, the endpoint was revision of the tibial and/or femoral component for aseptic loosening and/or osteolysis (except infection cases). The cumulative survival rate at the 10- and 15-year follow-up had no significant differences between the CR and PS groups (*p* = 0.239 and *p* = 0.352, respectively)^9^ (Fig. 2). Among the patients who were kept on follow-up, 12 in the CR group and 10 in the PS group presented complications (Table 4). There were three cases of infection in the CR group and two cases of infection in the PS group, which all required revision TKA. Aseptic loosening was found in seven patients from the CR group, with two cases of focal osteolysis. In the PS group, aseptic loosening was observed in three patients, with two cases of osteolysis (Fig. 3). However, one patient in the CR group refused to undergo implant revision. Of the two cases of osteolysis in the PS group, one was due to infection and one was isolated osteolysis. Additionally, one case of polyethylene wear and one case of quadriceps rupture were found in the PS group, with two cases of periprosthetic fracture in each group.

**Table 4:** Complications occurred in both groups until last follow up.

Parameters	CRF (101 knees)	PSF (170 knees)	*p*-value
Patients suffer from the complications	n = 12 (8.4%)	n = 10 (11.5%)	0.438*
Infection	3	2	
Aseptic loosening	7	3	
Osteolysis	2	4	
PE wear	0	1	
Quadriceps rupture	0	1	
Periprosthetic fracture	2	2	

*Chi-square test.

## Discussion

We found that the high-flexion CR and PS designs showed similar long-term outcomes in terms of ROM, HSS, KSS pain, KSS function, WOMAC score, and KSS radiographic outcomes. Moreover, the survival rate at 10 and 15 years showed no difference between the two designs.

While CR prosthesis in TKA provided paradoxical anterior motion of the femur, reducing the ROM^10^, the PS- prosthesis, creates a posterior roll back of the femur during flexion, increasing the ROM^11^. However, most of studies showed comparable results in ROM between the CR and PS designs in TKA , including our previous study^8,12^.

However, in the present study, the maximal flexion was similar between the two groups (CR: 131.1°, PS: 129.5°) after a 10-year follow-up and was comparable to the previously reported value^8,12^. On the contrary, a few studies have demonstrated that the CR TKA design has a more significant functional advantage than the PS TKA design, especially in high flexion^13^. This contradiction between biomechanical and functional outcomes suggests that the differences between the two groups may be either nonexistent or undetected by the current outcome measurements.

Although all clinical outcome parameters significantly improved after TKA with the use of either the CR or PS prosthesis, the clinical outcomes including the KSS, HSS score, and WOMAC score did not significantly differ between the two groups both preoperatively and postoperatively, similar to most other studies comparing CR and PS TKA^14,15^. In contrast, the other study reported better clinical outcomes with PS TKA than with CR TKA after 10 years follow-up^16^. They deduced that it was the degenerative change of PCL with time as a cause of worse outcomes in CR TKA… In recent studies on the clinical outcomes of TKA, the CR and PS TKA designs were shown to achieve similar clinical results. Several studies have compared the function of standard and high-flexion TKA designs, including our previous study^17,18^. Similar to the previous studies reporting no difference between the standard CR and PS knees in TKA, a few studies have shown the same clinical outcomes between high-flexion CR and PS prostheses in TKA after short-term follow-up. Kim et al. found no significant differences in KSS, HSS score, and patient satisfaction between the two groups^19^. However, another study reported that high-flexion CR TKA showed slightly better results than high-flexion PS TKA, the difference was not significant^13^.

The design of high-flexion prostheses differs in several major aspects from the design of standard prostheses. Major feature of high-flexion prostheses is the extend the radius and thickness of the posterior condyle of the femoral component to increase the contact area between femoral and tibial articulation during high flexion. However, Han et al. reported high the incidence of early aseptic loosening (38%) of high flexion PS knees in TKA at a mean of 23 months^4^. After then, early aseptic loosening, as a major concern of high-flexion prosthesis, has been previously investigated and reported.. They suggested that if deep knee flexion (ROM > 120°) is achieved and accompanied by migration of the femoral component, asymmetrical loading between the medial and lateral compartments of components of a total knee replacement may contribute to loosening and failure of the implant, which occurs at the implant-cement interface^4,5^. Similarly, Zelle et al. showed that during deep flexion (ROM > 120°), the shear stresses increased at the interface location beneath the proximal part of the anterior flange^5^. Cho et al. also reported an approximately 13.8% (30 of 218 knees) occurrence of a radiolucent line around the femoral component in high-flexion PS TKA, which might be related to passive maximal flexion activities, such as squatting or kneeling^20^. Most et al. also mentioned that asymmetrical loading between the medial and lateral compartments of components on deep knee flexion may contribute to loosening and failure of the implant^21^. However, a recent systematic review and long-term studies found no significant difference in the loosening or revision rate between high-flexion and standard TKA^22,23^. King and Scott reported only 15 loosening out of > 1600 knees after TKA, which might be related to inadequate surgical techniques including inaccurate surgical cuts, poor cementing technique, or deficient bone stock^24^. In the present study, 11 (6.9%) knees in the CR group and 10 (11.5%) knees in the PS group showed a radiolucent line in the femoral side, and 10 (6.3%) knees in the CR group and 6 (6.4%) knees in the PS group showed a radiolucent line in the tibial side. No significant differences were observed between the two groups. The present findings indicated that neither of the two high-flexion prostheses seemed to have caused a higher occurrence of a radiolucent line or loosening in the long-term follow-up results.

Multiple studies are steadily continuing with respect to the survival rate of the NexGen high-flexion system. Previous mid- to long-term follow-up studies showed satisfactory outcomes. Rhee et al. reported that NexGen LPS-Flex showed satisfactory outcomes with a 99.2% implant survival rate after 5 years^25^. Kim et al. also reported a 98.4% survival rate for NexGen LPS-Flex with a mean follow-up of 15 years^23^. Our study showed a similar implant survival rate. We documented successful results for total knee replacements with either a CR or a PS prosthesis. We focused on the correct flexion and extension gaps, well-balanced ligaments, and good cementing technique for a high success rate at 10 years.

However, our study had several limitations that should be considered. The first limitation is that this was not a randomized controlled study. Nevertheless, we adopted strict patient selection criteria and matched patients for body mass index, preoperative ROM, age, sex, and follow-up period (Table 1). Moreover, the study was also designed such that the same gap balance technique was used for the implantation of the CR and PS TKA components, except for either retaining or substituting the PCL and posterior tibial slope. Second, As the severely deformed knee is one of the indications for PS TKAs, the exclusion in this study might affect the clinical results in PS group. However, to compare the long-term follow-up between two groups, we had to exclude the patients with contraindications of CR TKAs and minimize the effects of pre-operative variables regarding postoperative clinical results. Third, the analysis of patients with a specific implant system (NexGen) might not be generalizable to patients with other arthroplasty systems. Nevertheless, the present study is the first to compare the long-term outcomes of high flexion CR and PS TKA prostheses.

## Conclusion

Similar to our hypothesis, we found no difference in terms of functional outcome, ROM, and complications, including the 10-year implant survival rate, between patients who received a high-flexion CR prosthesis and those who received a PS prosthesis during TKA. We conclude that the high-flexion CR and high-flexion PS knee designs provide similarly good ROM and clinical outcomes in the long term follow-up.
